# ID 69 - Revisão Rápida sobre Regorafenibe para Pacientes com Carcinoma Hepatocelular Avançado, de Baixo Risco e com Boa Capacidade Funcional em Segunda Linha de Tratamento

**DOI:** 10.5327/2237-9622.2025.v34s1.69

**Published:** 2025-11-25

**Authors:** Isabel Cristina de Almeida Santiago, Laura Augusta Barufaldi, Raphael Duarte Chança, Ricardo Ribeiro Alves Fernandes

**Keywords:** carcinoma hepatocelular, recidiva, regorafenibe, inibidores de proteínas quinases, revisão

## Abstract

**Introdução:**

Regorafenibe é um agente antineoplásico oral, inibidor da proteína quinase, recomendado como uma possibilidade de tratamento com a quimioterapia sistêmica paliativa de segunda linha do carcinoma hepatocelular (CHC) avançado ou metastático, quando os pacientes apresentarem boa capacidade funcional (escores ECOG 0 ou 1) e tiverem apresentado falha terapêutica ao uso de sorafenibe. Nesse contexto, foi realizada uma revisão rápida sobre o tema, com objetivo de auxiliar na tomada de decisão sobre a padronização do medicamento em um hospital de referência em oncologia.

**Método:**

Para responder se o regorafenibe é seguro e eficaz para o tratamento de segunda linha de pacientes com CHC, de baixo risco e com boa capacidade funcional, em 29 de maio de 2024 foram realizadas buscas nas bases de dados PubMed, Embase e Cochrane por revisões sistemáticas (RS) de ensaios clínicos randomizados (ECR). Esta revisão rápida foi conduzida de acordo com as recomendações da . O risco de viés das RS incluídas foi avaliado com a ferramenta (AMSTAR 2). A certeza geral das evidências para os desfechos sobrevida livre de progressão (SLP) e sobrevida global (SG) foi avaliada por meio do sistema (GRADE).

**Resultados:**

Ao final do processo, dez RS foram selecionadas. Todas apresentavam o mesmo e único ECR que comparava regorafenibe a placebo. Para extração dos desfechos, foram selecionadas quatro RS com metanálises em rede recentemente publicadas. A qualidade metodológica das quatro RS variou de "baixa" a "criticamente baixa". Por fim, optou-se pela apresentação dos resultados da mais recente, e cuja qualidade metodológica, não fosse inferior às das demais. As intervenções analisadas pela RS foram regorafenibe, cabozantinibe, ramucirumabe, pembrolizumabe, apatinibe e brivanibe, o comparador era placebo. A RS mostrou que todos os tratamentos prolongaram a SLP em comparação ao placebo. Para SG, no entanto, apenas regorafenibe (HR, 0,63; IC 95%, 0,5-0,79), cabozantinibe (HR, 0,76; IC 95%, 0,63-0,92), ramucirumabe (HR, 0,82; IC 95%, 0,7-0,96) e pembrolizumabe (HR, 0,79; IC 95%, 0,67-0,93) tiveram benefício. Já regorafenibe foi superior aos demais tratamentos em ganho de SG e o segundo mais eficaz em ganho de SLP (p-escore de 94,65% e 87,36%, respectivamente). Entretanto, esse benefício é discreto, pois o ganho proporcionado pelo regorafenibe em comparação ao placebo se restringe a algo entre 0,3 a 5,8 meses para SG e 1,2 a 2,8 meses para SLP. Quanto à segurança, regorafenibe teve a terceira maior incidência de eventos adversos (EA) grau ≥3 entre todos os tratamentos, porém, todas as comparações indiretas (CI) com regorafenibe não tiveram significância estatística. As CI com regorafenibe tiveram evidências variando de qualidade baixa a muito baixa, enquanto as evidências da comparação direta (CD) entre regorafenibe e placebo, eram de qualidade moderada.

**Conclusão:**

As conclusões das quatro RS selecionadas foram semelhantes, assim como suas limitações, destacando-se o poder limitado das metanálises em rede devido ao número limitado de CD. O regorafenibe mostrou-se seguro e eficaz no tratamento de segunda linha do CHC avançado, irressecável, de baixo risco, com boa capacidade funcional, após tratamento com sorafenibe. Apesar de regorafenibe ser provavelmente superior a outros tratamentos em ganho de SG, esse incremento limita-se a poucos meses.

